# Contrasting Roles of Mobile Genetic Elements and Metal Resistance Genes in Shaping the Gut Resistome of Wild Fish from the Qiantang River

**DOI:** 10.3390/ani16132000

**Published:** 2026-06-29

**Authors:** Yulai Dai, Yiqi Qiao, Nan Xie, Jinyong Zhu, Qicun Lin, Baoqing Xu, Yangxin Dai

**Affiliations:** 1 Hangzhou Academy of Agricultural Sciences, Hangzhou 310024, China; 2 School of Marine Sciences, Ningbo University, Ningbo 315211, China

**Keywords:** antibiotic resistance genes, mobile genetic elements, metal resistance genes, fish gut resistome, metagenomic assembly, Qiantang River

## Abstract

The dissemination of antibiotic resistance genes (ARGs) in riverine ecosystems poses a pressing public health threat, while the mechanisms governing the assembly of the gut resistome in wild fish remain poorly elucidated. This study aimed to elucidate the distributional patterns of ARGs across multiple environmental compartments and to identify factors associated with their variation, particularly the contributions of mobile genetic elements (MGEs) and metal resistance genes (MRGs) to gut resistome variation. Metagenomic sequencing was conducted on 60 samples, comprising water, sediment, and gut contents from three wild fish species (*Megalobrama terminalis*, *Aristichthys nobilis*, and *Coilia nasus*) with distinct feeding habits, collected from four reaches of the Qiantang River basin. A total of 305 ARG subtypes belonging to 23 classes were identified. ARG composition differed significantly across environmental media and host species (permutational multivariate analysis of variance, PERMANOVA; *p* < 0.01), with host species identity as the primary structuring factor. Variance partitioning analysis (VPA) revealed that MGEs independently explained the largest fraction of ARG variation in *A. nobilis* (33.8%, *p* = 0.006), whereas MRGs dominated in *C. nasus* (33.3%, *p* = 0.005); in *M. terminalis*, MGEs and MRGs together accounted for 47.9% of the variation. Metagenomic assembly recovered 2622 ARG-carrying contigs, of which 28.3% (743) were predicted as plasmid sequences; physical co-localization among ARGs, MGEs, and MRGs was detected on both chromosomes and plasmids. Metagenomic binning validated the physical co-localization of ARG-MGE-MRG modules in genera such as Morganella and Burkholderia at the genome level, while plasmid-borne high-risk ARGs were identified in Aeromonas. Risk ranking further revealed significant enrichment of Rank II potentially high-risk ARGs (e.g., *mcr-7.1*, *blaZ*) in fish guts, carried by potential pathogens. These findings suggest that horizontal gene transfer involving MGEs and co-selection related to MRGs are closely associated with the fish gut resistome composition in a manner dependent on host ecology, providing a scientific basis for shifting riverine resistance management from concentration-based control toward the interruption of dissemination pathways.

## Simple Summary

Antibiotic resistance in rivers is a growing global health threat. Resistant bacteria and genes can enter fish through polluted water and may eventually reach humans via the food chain, making it important to understand how these resistance genes form in fish guts. While many studies have examined resistance genes in river water and sediment, far less is known about their presence in wild fish intestines, which are environments where bacteria exchange genetic material easily. In this study, we collected water, sediment, and three wild fish species from the Qiantang River. The fish had different diets and habitats. We analyzed the antibiotic resistance genes in their intestines. We found the gene composition was closely tied to fish species, rather than the sampling location. Two main factors were associated with these patterns: mobile genetic elements (which help genes move between bacteria) and genes for heavy metal tolerance, though their roles varied among species. We also observed these genes tightly linked on DNA that can move between bacteria, suggesting they could spread. Our findings highlight that managing river resistance risks requires not only limiting antibiotic releases but also tackling heavy metal pollution and blocking gene transfer pathways.

## 1. Introduction

Antimicrobial resistance (AMR) is widely acknowledged as one of the foremost threats to global public health, causing an estimated 700,000 deaths annually [1]. This figure is projected to reach tens of millions by 2050 [2]. Within this escalating crisis, rivers function as critical conduits linking terrestrial pollution sources to aquatic ecosystems. They receive complex mixtures of antibiotics, heavy metals, and drug-resistant microorganisms from municipal sewage, industrial effluents, hospital discharges, and agricultural runoff. Consequently, these water bodies now constitute important environmental reservoirs and transmission pathways for antibiotic resistance genes (ARGs) [3,4,5]. In densely populated basins subject to intensive industrial and agricultural activities, the accumulation, evolution, and dissemination of ARGs pose persistent and far-reaching risks to aquatic ecological integrity and public health. The Qiantang River, one of the major waterways in eastern China, supports a high population density, a well-developed manufacturing sector, and intensive aquaculture, resulting in considerable anthropogenic pressure. Therefore, such conditions render this basin an ideal model system for investigating the environmental transmission of antibiotic resistance [6].

Against this background, substantial research has documented the occurrence, diversity, and dispersal of ARGs in riverine environments, establishing a critical knowledge base for understanding resistance pollution in watersheds [7,8,9]. However, existing investigations have predominantly targeted abiotic compartments, such as water and sediments, leaving the resistome within aquatic organisms, specifically the gut of wild fish, underexplored. As key consumers in river ecosystems, fish are directly exposed to environmental microorganisms and pollutants through gill respiration and feeding, while the gut itself constitutes a nutrient-rich microhabitat characterized by intense microbial interactions [10]. Previous work has confirmed that the fish gut provides a favorable niche for the conjugative transfer of mobile genetic elements (MGEs), particularly plasmids, and thus serves as a hub for ARG dissemination [11]. Traditionally, the fish gut resistome was thought to passively mirror environmental ARGs. However, new evidence suggests it acts as a selective filter and a recombination site for resistance genes.

Notably, the assembly of such a selective and recombination-active gut resistome is not simply an outcome of environmental exposure but is instead synergistically modulated by multiple biotic and abiotic factors. In the zebrafish gut, about 15% of fecal bacteria were found to acquire ARGs through RP4 plasmid-mediated conjugation, with the hindgut identified as the most active region for horizontal gene transfer [12]. The significant positive correlation between the integron *intI1* and the sulfonamide resistance gene *sul1* in aquaculture ponds, along with the tight coupling between MGE and ARG abundances in river ecosystems, collectively points to MGEs as the regulatory factors most intimately linked to ARG transmission [13,14]. Concomitantly, heavy metals have been shown to synergistically promote the maintenance and dissemination of ARGs through co-selection mechanisms [15]. Even in fishery sediments where antibiotic residues have fallen below detection limits, the persistence of certain ARGs may still be sustained by metals, highlighting the considerable role of metal–antibiotic co-selection pressure [16]. Furthermore, studies on fish from the Xiangjiang River basin and the coastal waters of Chile have identified opportunistic gut pathogens as potential hosts of ARGs, reinforcing the pivotal role of the gut environment in ARG transmission and risk assessment [17,18]. Based on these converging lines of evidence, we formulate three core hypotheses: (1) The fish gut resistome is not a passive mirror but shows strong host species specificity attributable to gut selection. (2) Horizontal gene transfer involving MGEs and co-selection related to metal resistance genes (MRGs) are key factors associated with the gut resistome, though their relative contributions may vary across fish species. (3) ARGs, MGEs, and MRGs cluster into genetic modules on chromosomes and plasmids, which may facilitate the horizontal dissemination of these genes.

However, a systematic dissection of the contributions of this co-regulatory model and its species-dependent variations among wild fish with different dietary habits and life-history traits is still lacking.

To address this knowledge gap, the present investigation selected the Qiantang River basin as a representative study area and chose four river sections spanning the upstream-to-downstream gradient, namely Jiande, Tonglu, Shangcheng, and Qiantang. Water, sediment, and gut samples of three dominant wild fish species with distinct feeding habits and life histories (*Aristichthys nobilis*, AN; *Coilia nasus*, CN; *Megalobrama terminalis*, MT) were simultaneously collected from these sections. Using metagenomic sequencing, the distribution of ARGs across multiple environmental compartments and among host species was characterized. Subsequently, multidimensional data on environmental factors, gut microbiota, MGEs, and MRGs were integrated, and variance partitioning and co-occurrence network analyses were employed to identify the key factors driving gut resistome variation and quantify their relative contributions. Furthermore, through metagenomic assembly and the genomic co-localization analysis, we sought evidence of physical co-occurrence of ARGs with MGEs and/or MRGs on the same contig to assess their potential for horizontal dissemination. Through this multi-tiered analytical framework, the present work aims to systematically elucidate the “mobile element-co-selection” co-regulatory system associated with the fish gut resistome, thereby providing critical scientific support for shifting resistance pollution management in river basins from conventional control of pollutant concentrations toward the precise interruption of dissemination pathways, and offering a theoretical reference for AMR risk mitigation in global rivers.

## 2. Materials and Methods

### 2.1. Sample Collection

**Figure 1:**
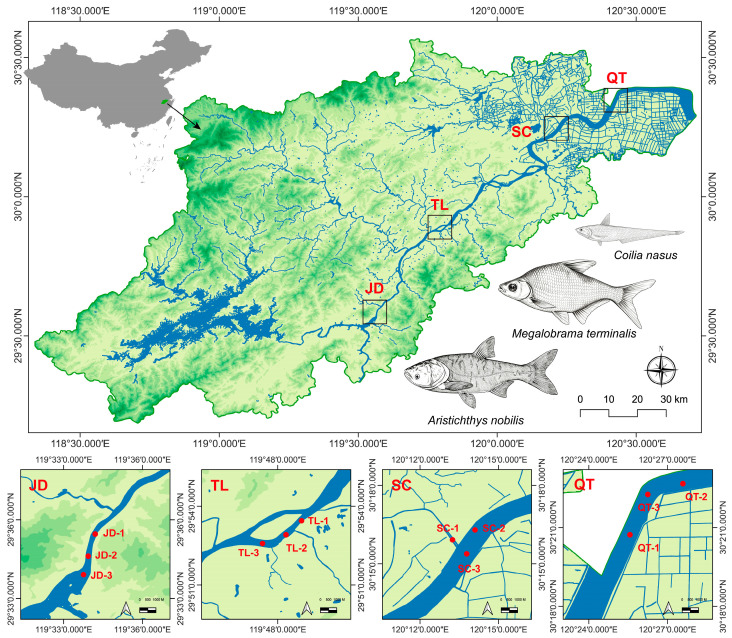
Sampling Sites. Schematic diagram showing the distribution of four representative sampling areas in the Qiantang River basin (Jiande JD1–3, Tonglu TL1–3, Shangcheng SC1–3, Qiantang QT1–3).

Four representative river sections in the Qiantang River basin, namely Jiande (JD), Tonglu (TL), Shangcheng (SC), and Qiantang (QT), were selected as sampling areas along the upstream-to-downstream gradient (Figure 1). The Qiantang River is located in Zhejiang Province, eastern China (28°04–30°25′ N, 117°37′–121°14′ E), and drains into the East China Sea. It is one of the major waterways in the region, sustaining a high-density population and intensive industrial and agricultural activities.

In August 2024, following the fishing ban period, surface water, sediment, and specimens of three dominant wild fish species (AN, CN, and MT) with distinct feeding habits and life histories were collected simultaneously from each sampling area. Three biological replicates were obtained for each sample type and area. For fish, the intestinal contents of each individual were treated as a separate biological replicate (3 individuals per species per area, totaling 36 fish across 4 areas × 3 species). No pooling of samples was performed, yielding a total of 60 independent samples (4 areas × 5 sample types × 3 replicates), thereby establishing a multi-compartment sampling system covering water, sediments, and fish guts.

Surface water samples (0.5 m below the surface) were collected using sterile water samplers (1 L per sample). After pre-filtration to remove large particles, microbial biomass was concentrated by vacuum filtration through 0.2 μm pore-size polycarbonate membranes (47 mm diameter; Millipore, Burlington, MA, USA). Surface sediments (0–5 cm) were collected using a grab sampler and placed in sterile self-sealing bags. Water and sediment samples were temporarily stored at low temperature under light-protected conditions. Fish were immediately immersed in an ice-water bath for anesthesia. Once locomotion ceased, they were euthanized by spinal cord transection using sterile sharp instruments prior to dissection. Intestinal contents were collected into 15 mL sterile cryovials and temporarily stored in a cooler with dry ice. All specimens were transported to the laboratory as quickly as possible for proper storage. All procedures involving wild fish in this study strictly complied with the Wildlife Protection Law of the People’s Republic of China and the relevant local regulations on fishery resource management in Zhejiang Province. No endangered or protected fish species were involved.

### 2.2. Determination of Physicochemical Parameters, Antibiotics, and Heavy Metals

Temperature, pH, and dissolved oxygen were measured in situ using a YSI 6000 Multiparameter Water Quality Sonde (YSI Inc., Yellow Springs, OH, USA). We determined nutrient parameters—including total nitrogen (TN), total phosphorus (TP), phosphate (PO_4_^3−^-P), ammonia nitrogen (NH_4_^+^-N), nitrite nitrogen (NO_2_^−^-N), and nitrate nitrogen (NO_3_^−^-N)—according to standard water quality analysis methods [19]. Antibiotics in water and sediment samples were extracted using a mixed solution of phosphate buffer (pH = 3.0), acetonitrile (1:1, *v*/*v*), and 0.1 g EDTA-2Na. We sonicated the samples for 30 min, centrifuged them at 12,000 rpm for 10 min, and repeated the extraction three times. The pooled extracts were analyzed by high-performance liquid chromatography–tandem mass spectrometry (HPLC-MS/MS) [20]. Metal elements were quantified using inductively coupled plasma mass spectrometry (ICP-MS) [21]. Detailed analytical performance parameters for antibiotic and heavy metal quantification, including limits of detection (LOD), limits of quantification (LOQ), and recovery ranges for all target analytes, are provided in Supplementary Table S1a,b.

### 2.3. DNA Extraction and Metagenomic Sequencing

Total genomic DNA was extracted from all 60 samples using the E.Z.N.A.^®^ Soil DNA Kit (Omega Bio-tek, Norcross, GA, USA) according to the manufacturer’s instructions. DNA concentration was measured with a Qubit^®^ 2.0 fluorometer (Life Technologies, Carlsbad, CA, USA), and DNA integrity was assessed by 1% agarose gel electrophoresis. We used the quality-checked DNA samples for metagenomic library construction and high-throughput sequencing. Briefly, we randomly fragmented approximately 1 μg of genomic DNA per sample using a Covaris S220 focused ultrasonicator (Covaris, Inc., Woburn, MA, USA) and recovered and purified fragments of approximately 450 bp for library preparation. All libraries were subsequently sequenced on the Illumina NovaSeq 6000 platform (Illumina, Inc., San Diego, CA, USA) in paired-end 150 bp mode, generating approximately 20 Gb of raw data per sample.

We subjected the raw sequencing reads to rigorous quality control. We used Trimmomatic (v0.39) [22] to remove adapter sequences and low-quality reads. For fish gut samples, we additionally used the BWA-MEM algorithm (parameters: -M -k 32 -t 16) to align quality-filtered reads to the corresponding fish reference genomes and eliminate host DNA contamination [23]. We retained the high-quality non-host reads (clean reads) for all downstream analyses.

### 2.4. Identification and Quantification of ARGs, MGEs, and MRGs

ARGs were annotated by aligning quality-controlled clean reads against the SARG database (v3.0) using BLASTX (v2.14.0) [24], with the following criteria: E-value ≤ 10^−7^, sequence similarity ≥ 80%, and alignment length ≥ 25 amino acids. ARG abundance was normalized to cell count and expressed as “copies per cell.”

MGEs were identified and quantified using the manually curated mobileOG-db (released 19 August 2022), and MRGs using the BacMet database [25], applying alignment thresholds of E-value ≤ 10^−10^, sequence similarity ≥ 80%, and coverage ≥ 70%. All gene abundances were normalized to cell count. To further account for differences in sequencing depth and gene length across samples, we additionally normalized gene abundances as transcripts per million (TPM), thereby enabling quantitative comparisons among samples. All subsequent statistical comparisons were performed using TPM-normalized values.

### 2.5. Metagenomic Assembly and Annotation

MEGAHIT (v1.2.9) was used to assemble clean reads from each sample into contigs [26]; only contigs ≥ 500 bp were retained for subsequent analysis. Open reading frames (ORFs) were predicted from the assembled contigs using Prodigal (v2.6.3) in metagenomic mode (-p meta) [27]. The predicted ORF amino acid sequences were aligned against the SARG database (v3.0) and the BacMet database (v2.0) using BLASTP (v2.14.0) to identify ARGs and MRGs [28,29], while ORF nucleotide sequences were aligned against the MGE database using BLASTN to identify MGEs [30]. The annotation filtering criteria were E-value ≤ 1 × 10^−5^, sequence similarity ≥ 60%, and alignment coverage ≥ 90%. The relaxed identity threshold (≥60%) was adopted to recover divergent homologues of ARGs, MGEs, and MRGs from assembled contigs, while the strict coverage requirement (≥90%) combined with the minimum contig length of 500 bp effectively excludes short or partial ORFs arising from assembly errors.

To investigate the horizontal dissemination potential of ARGs, contigs carrying at least one annotated ARG were defined as ARG-carrying contigs (ACCs). PlasFlow (v1.1.0) was used to predict the genetic localization of ACCs based on sequence signatures, classifying them as plasmid- or chromosome-derived or unclassified [31]. Concurrently, co-occurrence analysis of ARGs, MRGs, and MGEs was performed on ACCs. We considered an ARG- or MRG-carrying contig to possess the genetic structural basis for potential horizontal dissemination if PlasFlow predicted it as a plasmid sequence, or if an MGE was present within a 10-ORF window upstream or downstream.

### 2.6. Metagenomic Binning

To recover microbial genomes from the metagenomic assemblies, binning analysis was performed using assembled contigs. Contigs shorter than 1000 bp were removed first to improve binning reliability. Clean reads from each sample were then mapped back to the retained contigs using Bowtie2 to obtain contig coverage profiles across samples, and initial bins were generated using MetaBAT2 (v2.18) [32].

Bin quality was assessed using CheckM (v1.1.0) based on lineage-specific single-copy marker genes, with completeness and contamination estimated for each bin [33]. Bins with completeness > 50% and contamination < 10% were retained as metagenome-assembled genomes (MAGs) for subsequent analyses. The relative abundances of the retained MAGs were then quantified using CoverM (v0.7.0) [34].

The qualified MAGs were taxonomically assigned using GTDB-Tk (v2.4.0) [35]. To identify antibiotic resistance genes (ARGs), metal resistance genes (MRGs), and mobile genetic elements (MGEs) carried by the MAGs, open reading frames (ORFs) were predicted for each MAG using Prodigal (v2.6.3) [27]. The predicted amino acid sequences were searched against the SARG database (v3.0) [29] and the BacMet database (v2.0) [28] using BLASTP to annotate ARGs and MRGs, respectively. The corresponding nucleotide sequences were searched against a comprehensive MGE database (integrating mobileOG-db) [30] using BLASTN. The same filtering criteria used for functional gene annotation were applied here. Based on these analyses, the co-localization of ARGs, MRGs, and MGEs within individual MAGs was determined, providing genome-resolved links between resistance genes, metal resistance genes, mobile elements, and their microbial hosts.

### 2.7. Data Analysis

A schematic map of sampling sites was generated using QGIS (version 4.0; https://www.qgis.org), and all statistical analyses were conducted in R (version 4.3.3; https://www.r-project.org). The vegan package was used for principal coordinate analysis (PCoA) based on Bray–Curtis distances and permutational multivariate analysis of variance (PERMANOVA, permutations = 999). We performed Mantel tests using the ggcor package. We first calculated Euclidean distance matrices of environmental factors (antibiotic concentrations, heavy metal concentrations, physicochemical parameters, and ARG/MGE/MRG abundances) and then tested their correlations with the Bray–Curtis distance matrix of gut ARG communities using 999 permutations. We calculated Spearman’s rank correlations using cor.test. Raw *p*-values were adjusted with the Benjamini–Hochberg method (FDR-adjusted *p* < 0.05). Relative abundance matrices were filtered to keep only variables detected in at least 50% of samples from all groups. To account for zero inflation, we computed 95% bootstrap confidence intervals (1000 replicates) for each correlation coefficient. Heatmaps were generated with the ComplexHeatmap package. Significant associations were identified based on Spearman’s rank correlation coefficients (|*ρ*| > 0.7, *p* < 0.05) using the igraph package, and edges were further filtered by bootstrapping (1000 resamples) to construct co-occurrence networks and calculate topological parameters; optimized visualization was performed in Gephi (v0.10.1). Variance partitioning analysis (VPA) was carried out using the varpart function in vegan. Explanatory variables were pre-selected based on the results of Mantel tests (significant environmental factors, *p* < 0.05) and co-occurrence network analysis (hub nodes with the top 20% of degree values). Within each factor group (environmental factors, gut microbiota, MGEs, MRGs), principal component analysis (PCA) was first applied to reduce dimensionality, and the first principal component (PC1), which captured the largest proportion of variance within that group, was extracted as the representative variable and incorporated into the VPA model. For each species-specific model, *n* = 12 independent gut samples (4 reaches × 3 biological replicates). Adjusted *R*^2^ was reported to avoid overestimation of explanatory power; negative adjusted *R*^2^ fractions, which may arise from collinearity or represent values below random noise, were interpreted as zero. The significance of each independent fraction was tested by permutation (*n* = 999) using marginal effects from redundancy analysis (RDA), with the other three variable groups conditioned out. For the MAGs recovered from metagenomic binning, the co-localization of ARGs, MGEs, and MRGs within the same MAG was examined to provide genome-level evidence for their physical linkage. All ARGs were assigned risk ranks (Rank I–IV) following Zhang et al. [36].

## 3. Results

### 3.1. Distribution Characteristics of ARGs

**Figure 2:**
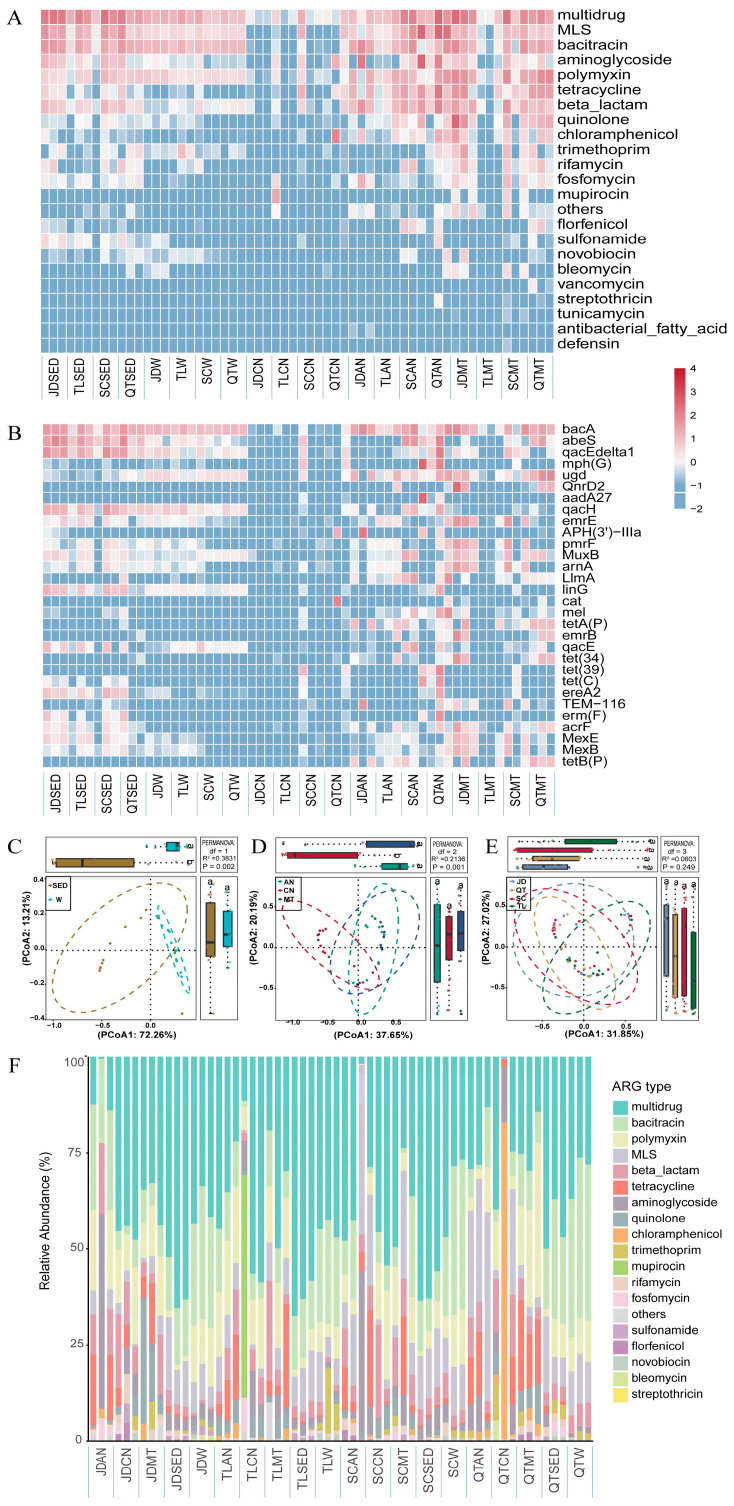
Distribution characteristics of ARGs at 12 sampling sites in the Qiantang River Basin. (**A**) Heatmap of the relative abundance of ARG types in each sample (log-transformed); (**B**) Heatmap of the top 30 ARG subtypes by relative abundance (log-transformed); (**C**) Principal Coordinate Analysis (PCoA) of ARG composition in sediments and water based on Bray–Curtis distance; (**D**) Principal Coordinate Analysis (PCoA) of ARG composition in the intestines of three fish species (AN, *Aristichthys nobilis*; CN, *Coilia nasus*; MT, *Megalobrama terminalis*) based on Bray–Curtis distance; (**E**) PCoA analysis of ARG composition across four sampling areas based on Bray–Curtis distance; (**F**) Composition proportions of the top 10 ARG types by relative abundance at each sampling site.

A total of 305 ARG subtypes belonging to 23 major classes were detected across the 60 samples from 12 sampling sites. The three most abundant ARG classes were multidrug resistance, MLS (macrolide-lincosamide-streptogramin), and bacitracin, while the most abundant subtypes were *bacA*, *abeS*, and *qacEdelta1* (Figure 2A,B). As shown in Figure 2C–E, ARG composition exhibited significant differences between sediment and water samples, as well as among different fish species (PERMANOVA based on Bray–Curtis distance, *p* < 0.01). Conversely, no significant differences were observed among the four sampling areas (*p* > 0.05). Further analysis of ARG class composition (Figure 2F) revealed that multidrug-resistant (MDR) ARGs accounted for the highest proportion in sediment, water, and MT samples, with average percentages of 57.08%, 38.30%, and 33.69%, respectively. Chloramphenicol resistance genes dominated in CN samples (average 37.29%), while MLS genes predominated in AN samples (average 28.67%).

### 3.2. Relationship Between ARGs and Environmental Factors

**Figure 3:**
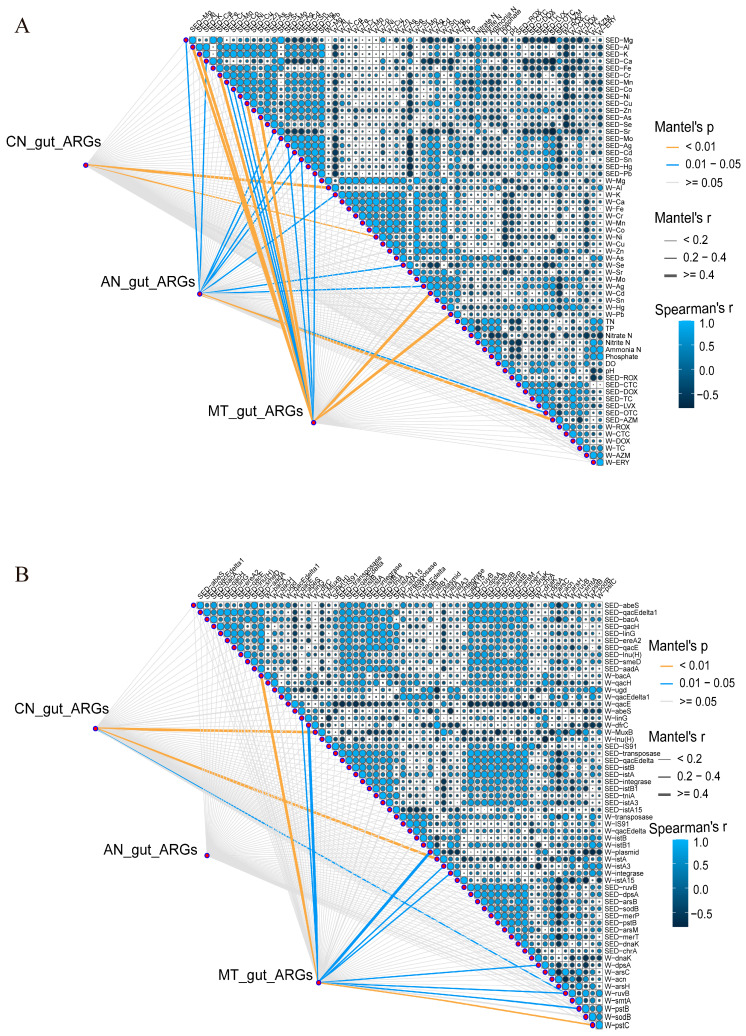
Mantel test of the relationship between the composition of intestinal ARGs in *Coilia nasus* (CN), *Aristichthys nobilis* (AN), and *Megalobrama terminalis* (MT) and environmental factors. (**A**) Correlation between gut ARG composition and antibiotics, heavy metals, and physicochemical parameters in water and sediments; (**B**) Correlation between gut ARG composition and ARGs, MGEs, and MRGs in water and sediments. Line width and color correspond to Mantel’s *r* statistic and significance level (*p*-value), respectively. A comprehensive table of all Mantel test results (*r* and *p*-values) is available in Supplementary Table S2.

To explore potential associations between environmental factors and gut ARG composition in AN, CN, and MT, Mantel tests were performed linking antibiotics, heavy metals, physicochemical parameters, and the top 10 ARGs, MGEs, and MRGs in water and sediments to gut ARG profiles. As illustrated in Figure 3A, gut ARG composition in the MT group exhibited highly significant positive correlations (Mantel’s *r* ≥ 0.4, *p* < 0.01) with multiple heavy metals, including Cu, Cr, Al, and K in sediments, as well as Cd and Pb in water. In the AN group, the strongest correlation was observed with azithromycin in sediments (*p* < 0.01), followed by sedimentary Ca and oxytetracycline, and water-borne Se and Ag (*p* < 0.05), indicating dual associations with both antibiotics and heavy metals, albeit with generally weaker correlation strengths than in MT. In the CN group, gut ARGs showed highly significant correlations only with Ni and Al in water (*p* < 0.01), with all other environmental factors failing to reach significance. Notably, no significant correlations were detected between gut ARGs and any water physicochemical parameters across the three groups. Figure 3B demonstrates that MT gut ARGs also exhibited highly significant or significant positive correlations with three ARG subtypes, three MGE classes, and four MRG subtypes in water samples, but not in sediments. In the CN group, gut ARGs were highly significantly correlated with W-*istA* and W-*MuxB* (*p* < 0.01), suggesting close associations with certain mobile elements and multidrug efflux pumps. For the AN group, Mantel test *p*-values between gut ARGs and the selected water and sediment-associated genes and mobile elements were all above 0.05.

### 3.3. Relationships Between Gut ARGs and MGEs, MRGs, and Microbial Communities

**Figure 4:**
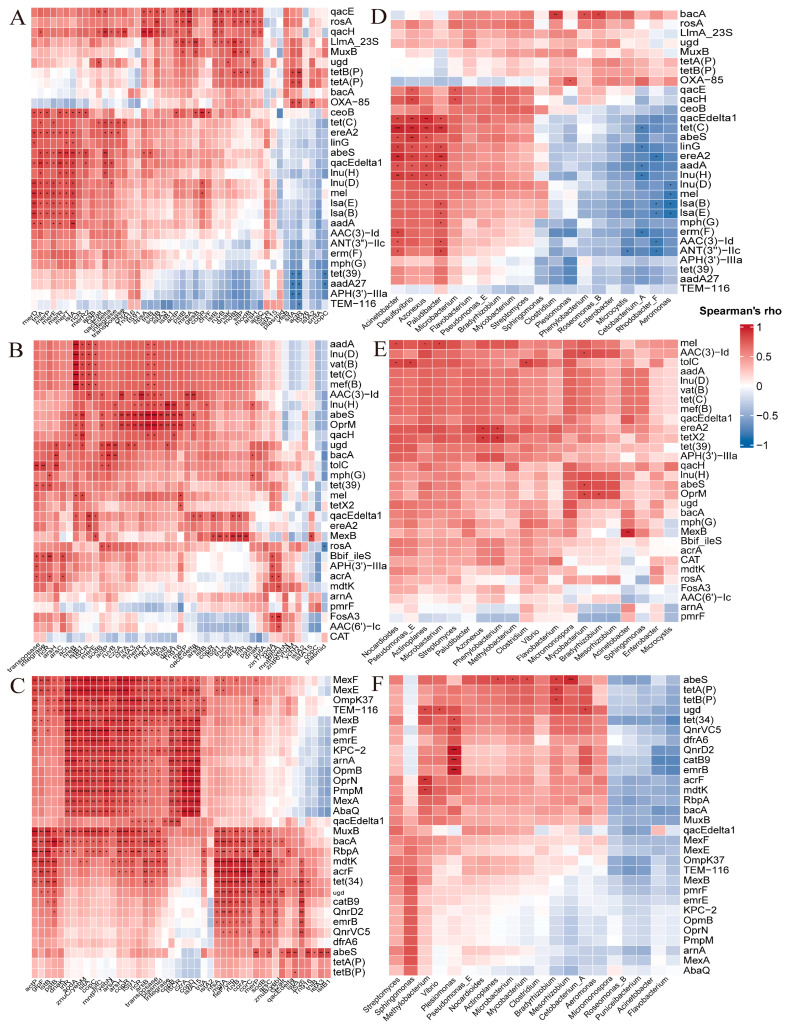
Heatmap of correlations between intestinal ARGs in the three fish species and MGEs, MRGs, and gut microbiota. (**A**–**C**) Spearman’s correlation analysis between intestinal ARGs from *Aristichthys nobilis* (AN), *Coilia nasus* (CN), *Megalobrama terminalis* (MT), MGEs and MRGs, respectively; (**D**–**F**) Spearman’s correlation analysis between intestinal ARGs from AN, CN, MT and the gut microbiota, respectively. Only correlations with FDR-adjusted *q* < 0.05 are shown. Heatmap colors represent positive (red) or negative (blue) correlation coefficients. Significance levels based on FDR-adjusted *q*-values: * *q* < 0.05, ** *q* < 0.01, *** *q* < 0.001. The complete correlation matrix data are available as Supplementary Table S3.

Spearman correlation analysis was performed between the top 30 gut ARGs, all detected MGEs, the top 30 MRGs, and the top 20 gut microbial genera in each fish species (Figure 4A–F). After FDR correction (*q* < 0.05), a considerable number of significant correlations were identified in each group, with pairs exhibiting extremely strong positive correlations (*ρ* > 0.8) accounting for a notable proportion, indicating the existence of dense co-occurrence networks among ARGs, MGEs, MRGs, and the gut microbiota. It is important to note that these co-occurrence patterns are based on statistical correlations and should not be interpreted as direct evidence of physical linkage or shared hosts.

In the AN group, many ARGs showed strong, highly significant positive correlations with MGEs (*ρ* > 0.8, *q* < 0.01), including *tniB*, *istA*, and *tniA*. These ARGs also correlated strongly and positively with MRGs (*ρ* > 0.8, *q* < 0.01), such as *dpsA*, *mreA*, and *copR*. These results suggest that heavy metal–antibiotic co-resistance mechanisms may help maintain these associations. In the CN group, we observed stronger evidence of physical linkage. Genes like *tet(C)*, *mef(B)*, and *vat(B)* co-occurred perfectly with the MRG *nreB* (nickel resistance protein NreB) (*ρ* = 1.0, *q* < 0.001), a pattern highly suggestive of physical proximity on the same genetic element, though contig-level validation is required to confirm this. Furthermore, *merT* and *furA* were highly correlated with nearly all major ARGs in this group (*ρ* > 0.8, *q* < 0.001), suggesting that mercury and iron regulation may act as important co-selective factors. In the MT group, *AbaQ* (multidrug efflux pump AbaQ) also showed perfect co-occurrence with the heavy metal resistance genes *czrA* and *copD* (*ρ* = 1.0, *q* < 0.001). While such perfect correlations may reflect physical proximity, this inference remains correlational and requires further genomic structural validation. Mobile genetic elements associated with heavy metal resistance (Cu, Zn, Co, Cd, Hg, etc.) correlated almost entirely with antibiotic/disinfectant resistance genes (ARGs), suggesting a statistical association between environmental heavy metal contamination and antibiotic resistance patterns. Whether this reflects co-selection or physical co-transfer requires further investigation.

Regarding the relationship between gut ARGs and microbial communities, interspecies differences were also clear. In the AN group, *Acinetobacter* showed highly significant positive correlations (*q* < 0.01) with several ARGs, including *tet(C)*, *ereA2*, and *abeS*, while *Desulfovibrio*, *Paludibacter*, and *Azonexus* showed significant positive correlations (*q* < 0.05) with genes such as *abeS*, *qacEdelta1*, and *tet(C)*. In the CN group, *Acinetobacter* correlated highly significantly and positively with *MexB* (*q* < 0.01), whereas *Mycobacterium* correlated significantly and positively with *abeS*, *AAC(3)-Id*, and *OprM* (*q* < 0.05). In the MT group, *Plesiomonas* displayed a perfect positive correlation with *QnrD2* (*ρ* = 1.0, *q* < 0.001) and significant positive correlations with *emrB*, *catB9*, *QnrVC5*, and others (*q* < 0.05); *Sphingomonas* was strongly and positively correlated with ARGs such as *emrE*, *pmrF*, and *arnA* (*q* < 0.05). Notably, *Acinetobacter* showed strong correlations with diverse ARGs across multiple groups, while *Plesiomonas*, *Sphingomonas*, and others also stood out in their respective groups, suggesting that these genera may be important potential hosts of resistance genes.

The potential host bacteria identified above are inferred solely from co-occurrence correlations. Such correlations do not constitute definitive proof of direct carriage, as they may also arise from shared environmental preferences or syntrophic interactions among bacterial taxa.

**Figure 5:**
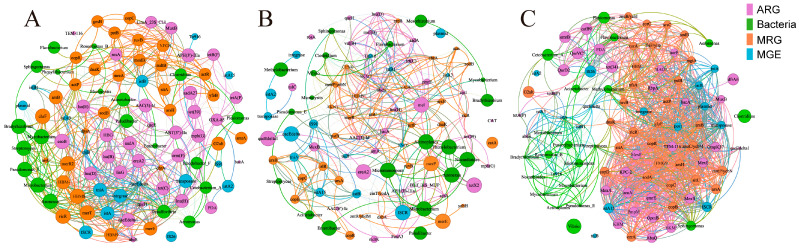
Co-occurrence network of ARGs, MGEs, and MRGs in the intestines of three fish species and the intestinal microbiota. (**A**) Co-occurrence network in the *Aristichthys nobilis* (AN) gut; (**B**) Co-occurrence network in the *Coilia nasus* (CN) gut; (**C**) Co-occurrence network in the *Megalobrama terminalis* (MT) gut. Nodes represent ARG subtypes, MGEs, MRGs, and microbial genera. Edges indicate significant associations based on Spearman’s correlation coefficient (|*ρ*| > 0.7, *p* < 0.05) and validated by bootstrapping (1000 resamples). Node size is proportional to degree, and node color distinguishes different gene or microbial categories. To better identify key nodes, the top 10 hub nodes by degree in each panel are listed here: for AN, [*tniA*, *linG*, *qacEdelta*, *ereA2*, *istA*, *merT*, *Desulfovibrio*, *merR*, *lnu(H)*, *merR2*]; for CN, [*abeS*, *OprM*, *istB1*, *merT*, *furA*, *mntP/yebN*, *istB*, *mel*, *dpsA*, *czcA*]; for MT, [*IS91*, *TEM-116*, *copR*, *arsH*, *irlR* (iron-lead resistance regulator), *ricR*, *sitA*, *MexF*, *glpF*, *znuC/yebM*].

Co-occurrence networks were further constructed using the bootstrap method (threshold |*ρ*| ≥ 0.7) (Figure 5A–C). In the AN network, the top 10 hub nodes by degree comprised 2 ARG types, 3 MGE types, 1 MRG type, and 1 bacterial genus, all with degree values ≥ 23, demonstrating a high degree of co-occurrence. MGE nodes occupied the most central positions; ARG hubs were all lincosamide and macrolide resistance genes; the three MRG hubs covered mercury transport (*merT*) and transcriptional regulation (*merR*) functions; *Desulfovibrio*, the sole microbial hub, exhibited significant co-occurrence with multiple ARG and MGE nodes. In the CN network, the top 10 hubs comprised 2 ARG types, 1 MGE type, and 4 MRG types, with no bacterial genera present. Efflux pump genes represented the largest proportion of ARG hubs, and the selected MRGs performed highly diverse functions. The MT network displayed extremely high connectivity and clustering (all top 10 degree values ≥ 44). The top 10 hubs consisted of 2 ARG types, 1 MGE type, and 6 MRG types, with no bacterial genera. Among them, *IS91* occupied the absolute core of the network with the highest degree (50), followed by *TEM-116* (48), exhibiting high co-occurrence of multi-metal and multi-pathway resistance.

### 3.4. Factors Associated with Variation in Gut ARG Distribution

**Figure 6:**
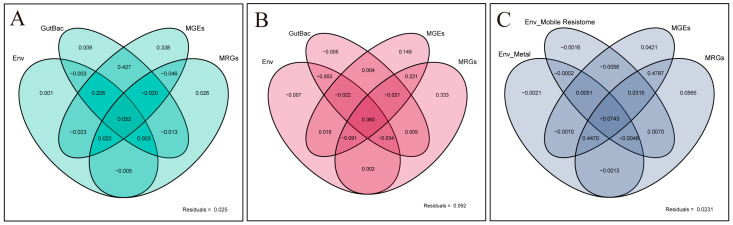
Variance decomposition analysis (VPA) of ARG composition variation in the intestines of the three fish species. (**A**) VPA analysis of ARGs in the gut microbiota of *Aristichthys nobilis* (AN); (**B**) VPA analysis of ARGs in the gut microbiota of *Coilia nasus* (CN); (**C**) VPA analysis of ARGs in the gut microbiota of *Megalobrama terminalis* (MT). The explanatory variable groups included environmental factors, gut microbiota, MGEs, and MRGs, with environmental factors encompassing relevant indicators such as environmental metals and environmental resistance profiles. The values in each Venn diagram represent the independent or combined explanatory power (*R*^2^) of each factor group on ARG variation. Negative adjusted *R*^2^ values may arise from collinearity among explanatory variables or represent explanatory power below random noise. In the statistical assessment, such values were treated as zero.

To quantitatively assess the relative contributions of environmental factors, gut microbiota, MGEs, and MRGs to the overall variation in gut ARGs, significant environmental factors identified by Mantel tests and key nodes from co-occurrence networks were selected as explanatory variables. Variance partitioning analysis (VPA) with permutation tests was subsequently employed (Figure 6A–C).

Prior to VPA, PCA was applied to each variable group, and PC1 was extracted as the representative variable. In *A. nobilis*, PC1 explained 68.3% (environmental factors), 71.9% (gut microbiota), 97.8% (MGEs), and 75.0% (MRGs), with ARGs at 50.3%. In *C. nasus*, the values were 67.9%, 59.0%, 62.3%, and 75.9%, with ARGs at 84.3%. In *M. terminalis*, where environmental factors were divided into metals and resistome groups and gut microbiota was not included, PC1 explained 75.3% (environmental metals), 51.5% (environmental resistome), 71.6% (MGEs), and 90.1% (MRGs), with ARGs at 76.5%.

In the AN group, MGEs showed a significant independent explanatory effect (adjusted *R*^2^ = 0.338, *p* = 0.006), representing the largest independent contribution among all factors. MRGs exhibited a smaller but significant independent effect (adjusted *R*^2^ = 0.026, *p* = 0.019). The independent effects of environmental factors (adjusted *R*^2^ = 0.001, *p* = 0.276) and gut microbiota (adjusted *R*^2^ = 0.009, *p* = 0.123) were not significant. The full model was significant (*p* = 0.004). In the CN group, MRGs provided the strongest independent explanatory effect (adjusted *R*^2^ = 0.333, *p* = 0.005), followed by MGEs (adjusted *R*^2^ = 0.149, *p* = 0.015). Environmental factors and gut microbiota showed no significant independent contributions (adjusted *R*^2^ < 0, interpreted as zero; *p* = 0.566 and 0.447, respectively). The shared fraction between MGEs and MRGs accounted for an additional adjusted *R*^2^ of 0.221. The full model was significant (*p* = 0.026). In the MT group, MGEs and MRGs together accounted for 47.9% of the total variation (adjusted *R*^2^ of shared fraction [j] = 0.479), the highest combined explanatory power among the three species. Both MGEs and MRGs showed significant independent effects (adjusted *R*^2^ = 0.042, *p* = 0.006 and adjusted *R*^2^ = 0.056, *p* = 0.004, respectively), while environmental factors had negligible independent contributions (adjusted *R*^2^ < 0, interpreted as zero; *p* = 0.629 and 0.571). The full model was highly significant (*p* = 0.001).

These statistical associations suggest that MGEs and MRGs are the factors most closely associated with gut ARG variation, though the correlational nature of this evidence does not establish direct causation. We should clarify that this joint fraction in VPA represents shared variation due to collinearity between the two factor groups, not a statistically verified biological synergistic interaction. Environmental factors and gut microbiota did not show significant independent explanatory power across the three species, likely because their effects overlap substantially with those of MGEs and MRGs.

### 3.5. Genetic Co-Localization and Horizontal Dissemination Potential of ARGs, MRGs, and MGEs

To validate the physical linkage between ARGs and MGEs/MRGs and their horizontal dissemination potential at the genomic level, co-localization analysis was performed on metagenomic assemblies. A total of 2622 ACCs carrying 2952 ARG-like ORFs were identified. PlasFlow predicted 743 ACCs (28.3%) as plasmid sequences, 1124 (42.9%) as chromosome-derived, and 755 (28.8%) as unclassified, indicating that a considerable fraction of these ARGs may reside on mobile genetic elements. A complete list of all 2622 ACCs with detailed annotations, including contig ID, contig length, ARG/MGE/MRG annotations, ORF positions, and PlasFlow-predicted localization, is provided in Supplementary Table S4.

**Figure 7:**
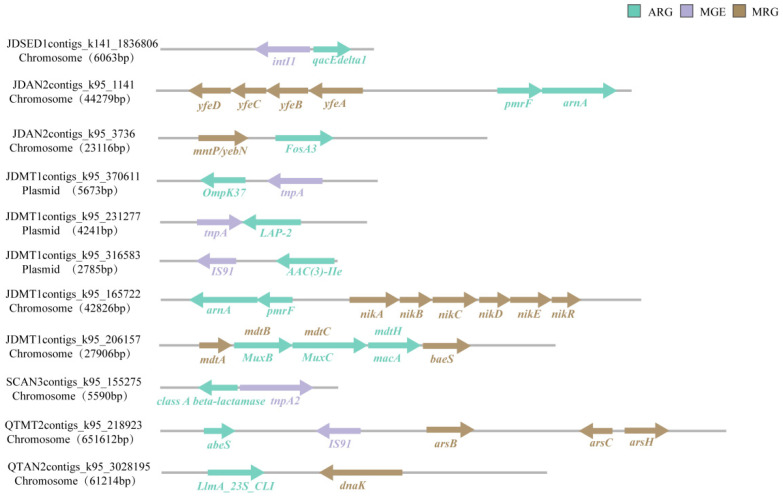
Co-localization analysis of ARGs, MRGs, and MGEs on metagenomic assembly contigs. The network diagram illustrates the physical co-occurrence of ARGs, MRGs, and MGEs on Chromosome and Plasmid sequences. Edges indicate physical linkage between an ARG/MRG and an MGE on the same contig, where the ARGs are within 10 adjacent open reading frames (ORFs). Node colors distinguish different classes of genetic elements (ARGs, red; MRGs, blue; MGEs, green). The detected ARGs include multidrug (*qacEdelta1*, *abeS*, *MuxB*, *MuxC*, *mdtH*), fosfomycin (*FosA3*), beta-lactam (*OmpK37*, *LAP-2*, *class A beta-lactamase*), aminoglycoside (*AAC(3)-IIe*), polymyxin (*arnA*, *pmrF*), and macrolide-lincosamide-streptogramin (*LlmA_23S_CLI*, *macA*); MGEs include integrase (*intI1*), transposase (*tnpA*, *tnpA2*), and insertion sequence (*IS91*); MRGs include Iron (*yfeD*, *yfeC*, *yfeB*, *yfeA*), Manganese (*mntP/yebN*), Nickel/Cobalt (*nikA*, *nikB*, *nikC*, *nikD*, *nikE*, *nikR*), Multimetal (*mdtA*, *mdtB*, *mdtC*, *baeS*), Arsenic (*arsB*, *arsC*, *arsH*), and General stress response (*dnaK*). The complete contig-level evidence for all 2622 ARG-carrying contigs is available in Supplementary Table S4.

Co-occurrence analysis of ARGs, MRGs, and MGEs on ACCs (Figure 7) revealed that the chromosomal integrase *intI1* co-occurred with *qacEdelta1*, and the transposase *tnpA2* co-occurred with *class* A *β-lactamase* genes, forming ARG-MGE linkages. On chromosomes, multidrug efflux MRG clusters (*mdtA*, *mdtC*, *baeS*, etc.) coexisted with multidrug ARG clusters (*MuxB*, *MuxC*, *mdtH*, etc.), constituting ARG-MRG combinations. On plasmids, ARG-MGE co-occurrences such as *IS91* and *AAC(3)-IIe* (aminoglycoside N-acetyltransferase AAC(3)-IIe) were observed, along with ARG-MRG combinations where *pmrE* and *arnA* were co-localized with the nickel transport MRG cluster *nikA-nikR*.

### 3.6. Genomic Hosts of ARGs and Co-Localization with MGEs and MRGs

A total of 193 quality-checked MAGs were recovered. Among them, 80 MAGs harbored at least one ARG. Of these ARG-carrying MAGs, 58 also contained MRGs, 25 contained MGEs, and 19 contained all three (Supplementary Table S5). For example, a *Morganella* sp. affiliated with Pseudomonadota carried genes conferring resistance to polymyxin (*arnA*, *pmrF*), multidrug (*mdtK*, *emrB*, *MuxB*), tetracycline (*tet(34)*), beta-lactam (*DHA-1*), and bacitracin (*bacA*), alongside multiple MGEs and MRGs, with some genes located on plasmid sequences. Other clinically high-risk ARGs were also identified. The polymyxin resistance gene *mcr-7.1* was carried by an *Aeromonas* MAG on a plasmid, together with multiple MRGs but no annotated MGEs, suggesting potential for horizontal dissemination via plasmid transfer rather than direct MGE-mediated co-localization. The beta-lactam resistance gene *blaZ* was hosted by a *Staphylococcus* MAG. These results provide direct genomic evidence for the physical co-existence of ARGs, MGEs, and MRGs within specific hosts such as *Morganella* and *Burkholderia*, while plasmid-borne high-risk ARGs were identified in other genera. This confirms some of the potential hosts inferred by co-occurrence networks, but it also demonstrates that not all network correlations reflect physical linkages, highlighting the necessity of binning validation.

### 3.7. Distribution of Clinically High-Risk ARGs

Based on the risk ranking framework, Rank I ARGs (e.g., *sul1*, *qacEdelta1*, *tet(M)*) were detected across all compartments, while Rank II ARGs (e.g., *mcr-7.1*, blaZ, *OXA-347* (OXA-type carbapenemase), *AAC(3)-Ia*) were found exclusively or predominantly in fish gut samples, particularly in *M. terminalis* and *A. nobilis*. Notably, *mcr-7.1* was exclusively detected in *A. nobilis* gut samples on a plasmid sequence, and *blaZ* was found in multiple fish guts but absent from water. Metagenomic binning (Section 3.6) further confirmed that *mcr-7.1* was carried by an *Aeromonas* MAG on a plasmid, providing genome-level evidence for its mobility potential, while *blaZ* was hosted by a *Staphylococcus* MAG. These results identify wild fish guts as important reservoirs of potentially clinically high-risk ARGs, with specific bacterial hosts and genetic contexts that may facilitate their dissemination.

## 4. Discussion

To our knowledge, the present investigation represents the first integrated metagenomic survey in the Qiantang River basin to simultaneously characterize the gut, water, and sediment resistomes of multiple wild fish species, delineating the distributional patterns of gut ARGs and the factors associated with their variation. The observations demonstrate that while the composition of the fish gut resistome displays pronounced species specificity, its underlying associative patterns are highly consistent: MGEs and MRGs are closely associated with the majority of gut resistome variation. These statistical associations suggest that horizontal gene transfer and metal–antibiotic co-selection may play important roles in shaping the resistome, though the correlational nature of this evidence does not establish direct causation. These findings depart from the traditional emphasis on either “environmental exposure” or “microbiota composition” as primary determinants of ARG profiles [37,38]. Instead, they suggest an explanatory model in which horizontal gene transfer involving MGEs and co-selection related to MRGs play central roles in understanding the distribution of ARGs in wild fish guts.

### 4.1. Distribution Characteristics and Host Specificity of Gut ARGs

In the current work, a total of 305 ARG subtypes were detected across the guts of three fish species, along with water and sediments, covering 23 major ARG classes. Multidrug resistance, MLS, and bacitracin types predominated, with *bacA*, *abeS*, and *qacEdelta1* as core subtypes, a pattern consistent with prevailing reports from global aquatic ecosystems [39,40,41,42]. PCoA and PERMANOVA revealed highly significant differences among fish species and between water and sediment (*p* < 0.01), but not among regions (*p* > 0.05), underscoring host species identity as the primary factor shaping gut resistome structure [43]. The predominance of multidrug resistance genes in MT, chloramphenicol resistance genes in CN, and MLS genes in AN contrasted markedly with the MDR-dominant profiles of water and sediment. Consequently, these results indicate that ARGs are substantially reshaped upon entering the gut, consistent with the notion that the fish gut functions as a selective filter and amplifier for ARGs rather than a mere passive carrier [44,45].

### 4.2. Differential Responses of Gut ARGs to Environmental Selection Pressures

Mantel tests indicated divergent responses of gut ARGs to environmental selection pressures among the three species. The strong correlations between MT gut ARGs and sediment Cu, Cr, and Al, as well as water Cd and Pb, together with their close associations with water-borne ARGs, MGEs, and MRGs, align with the positive selection of ARG-hosting bacteria by heavy metals reported previously [46]. The dual associations of AN gut ARGs with azithromycin, oxytetracycline, and several heavy metals are consistent with the co-selective maintenance of ARGs by metals and antibiotics [16]. Conversely, the limited sensitivity of CN gut ARGs to environmental stressors may reflect their higher trophic position and migratory lifestyle. Notably, the absence of significant correlations between gut ARGs and water physicochemical parameters (e.g., TN, TP, ammonia nitrogen) across all groups suggests that heavy metals or antibiotics, rather than conventional nutrient variables, are the environmental factors most intimately linked to gut resistome variation [47,48]. This observation parallels the finding that Astragalus polysaccharides influence ARGs primarily through modulating the microbial community and MGEs rather than through direct chemical concentration effects [49], further supporting the inference that ARG presence in the gut is more intimately associated with biological mediators (host bacteria, MGEs) and co-selection pressures than with simple chemical exposure.

### 4.3. Co-Selection and Co-Transfer Networks of ARGs-MGEs-MRGs and the Gut Microbiota

The prevalence of strong positive correlations (*ρ* > 0.8, *p* < 0.01) and highly connected co-occurrence networks observed across all three fish species provides corroborative evidence that co-selection and horizontal gene transfer are likely major mechanisms for ARG dissemination in the fish gut. These findings are consistent with previous reports demonstrating that MGE abundance is strongly positively correlated with ARG abundance and that MGEs represent the factors most closely associated with ARG transmission [13,50,51].

In the AN group, MGEs such as *istA*, *integrase*, and *tniA* formed strong associations with MRGs (*merT*, *merR*) and multiple ARGs, with *Desulfovibrio* emerging as a key hub. The role of specific bacterial genera as ARG hosts has also been highlighted in other wild fish studies; for instance, *Pseudomonas* was identified as a major carrier of clinically important β-lactam and phenylpropanol ARGs in the gut of Chilean marine fish [18], further supporting the critical role of specific gut microbiota in ARG enrichment and transmission. In the CN group, the extremely strong co-occurrence of *tet(C)* and *mef(B)* with *merT* and *furA* (*ρ* = 1.0) posits a possible tight physical linkage, with mercury/iron metabolism systems serving as potential co-selection factors. In the MT group, *IS91* occupied the absolute core of the network with the highest degree (50), indicating that insertion sequence-mediated gene capture and transposition may have played a significant role. This finding is akin to observations that *IS* family transposases were found to be among the abundant MGE types in the Bahe River water microbiome and co-exist with various ARGs on plasmids [40]. Co-occurrence networks revealed strong associations of ARGs and MGEs with genera such as *Desulfovibrio*, *Acinetobacter*, and *Plesiomonas*. Binning validated several of these at the genome level—*Morganella* and *Burkholderia* carried diverse ARG-MGE-MRG modules, while *Aeromonas* harbored the high-risk gene *mcr-7.1* on a plasmid alongside MRGs (Section 3.6). *Acinetobacter*, though lacking detected MGEs in its MAGs, harboured multiple ARGs and MRGs, consistent with its central role in the co-occurrence network. However, some correlations (e.g., *Desulfovibrio*-ARG) lacked MAG confirmation, likely reflecting strain-level dispersal or incomplete binning. Host inferences from networks should therefore be interpreted alongside binning evidence. Notably, risk ranking revealed that Rank II ARGs, including the mobile colistin resistance gene *mcr-7.1* and β-lactamase gene *blaZ*, were detected exclusively or predominantly in fish gut samples. Binning confirmed that *mcr-7.1* was carried by *Aeromonas* on a plasmid, providing a structural basis for horizontal dissemination. The detection of the carbapenemase gene *OXA-347* further suggests overlap between environmental and clinical resistomes. These findings underscore the importance of incorporating risk ranking into routine surveillance, with particular attention to fish guts as potential convergence points for pathogens and high-risk genes under the One Health framework.

### 4.4. Associative Patterns of the Resistome with MGEs and MRGs as Key Explanatory Factors

VPA in the three wild freshwater fish species quantitatively confirmed that MGEs and MRGs constitute the two core explanatory factors for gut ARG variation; however, their combinatorial patterns exhibited pronounced interspecific differences, which can be explained by species-specific dietary habits, habitats, and physiological characteristics.

As a filter-feeding pelagic fish [52], AN ingests plankton and suspended organic debris, taking up large quantities of free-living bacteria and DNA from the water column. Previous work has shown that antibiotic resistance genes can be transferred from manure-contaminated water to the gut microbiota of filter-feeding fish along the food chain [53]. Accordingly, the dominant explanatory power of MGEs (33.8%) suggests that water-derived mobile elements may undergo frequent horizontal dissemination within the gut microbiota through mechanisms such as transformation or transduction, with filter-feeding acting as a continuous “inoculation” process that renders horizontal gene transfer the predominant pathway for ARG dissemination. In contrast, CN is a migratory piscivorous fish occupying a higher trophic level [54]. Studies have demonstrated that this species exhibits a significantly higher capacity for heavy metal accumulation compared to other carnivorous fish at the same trophic level [55]. The predominance of MRGs in explaining its gut ARG variation (33.3%) suggests as a plausible interpretation, that long-term metal selection pressure—potentially mediated by dietary metal uptake—may favour the integration of ARGs into chromosomes and the maintenance of resistance traits via coselection. In this context, MGEs would play a more subsidiary role. Likewise, MT, an omnivorous mid-to-lower-water-layer fish that feeds by disturbing bottom sediments, has been classified as a benthic omnivore [56]. Through this feeding behaviour, it is continuously exposed to heavy metals and diverse MGEs in sediments. The nearly equal combined explanatory power of MGEs and MRGs (47.9%) reveals a strong statistical association, consistent with the intertwined biological processes of co-selection and co-transfer.

Taken together, these interspecific differences essentially reflect species-specific niche differentiation. The feeding habits and water-column habitats of fish determine their modes of exposure to environmental pollutants and microorganisms, thereby contributing to their unique resistome profiles and associative patterns [38].

### 4.5. Evidence from Genomic Co-Localization

Metagenomic assembly and contig co-localization analysis provide direct structural support for the physical linkage and horizontal potential for horizontal gene transfer of ARG-MGE-MRG units at the genomic level. Plasmids are important vectors for the potential horizontal dissemination of ARGs; in this study, 28.3% of assembled ACCs were predicted as plasmid sequences. This observation corroborates the high plasmid carriage rate (53.5%) previously observed in antibiotic-resistant bacteria isolated from wild fish guts, collectively suggesting that the fish gut provides a favorable environment for plasmid-mediated conjugative transfer, thereby accelerating ARG dissemination [18]. On chromosomes, *intI1* forms an ARG-MGE linkage with *qacEdelta1*, and *tnpA2* with β-lactamase genes; the mdt efflux cluster forms an ARG-MRG co-localization structure with multidrug ARGs [40]. Such physical linkages at the genomic level constitute key evidence that ARGs are transmitted across hosts via horizontal gene transfer. For example, in *Aeromonas hydrophila* from freshwater fish, *tmexCD3-toprJ1* and *blaCTX-M-3* were found within novel genomic islands captured by *Tn6855* variants and *In4*-type integrons, respectively, structures that confer high transfer potential [57]. These co-localized structures indicate that ARGs, MGEs, and MRGs in the fish gut of the Qiantang River basin may constitute genetic modules for synergistic transmission, which may facilitate their horizontal dissemination via plasmids, transposons, and integrons and thereby substantially elevating the risk of resistance dissemination in aquatic ecosystems. Furthermore, the genomic physical linkage evidence suggests that MGEs, particularly integrons and transposons, are not only key factors explaining resistome variation but also hold promise as early warning indicators for resistance transmission risks in river basins, potentially offering greater monitoring value than simply tracking specific ARG subtypes.

Importantly, these physical linkage patterns at the genomic level provide direct structural support for the VPA-derived associative model, in which MGEs and MRGs jointly shape the gut resistome. The co-localization of ARGs with MGEs on plasmids and with MRGs on chromosomes provides physical evidence to support the statistical inference, suggesting that horizontal gene transfer and metal co-selection are among the major factors associated with gut resistome assembly.

### 4.6. Limitations and Future Directions

This study has two limitations. First, samples were collected only in August 2024, representing a single season. Seasonal environmental fluctuations may alter ARG, MGE, and MRG profiles, thereby limiting the generalizability of host-driven resistome patterns. Second, the key associations among ARGs, MGEs, and MRGs reported here are fundamentally correlational. The statistical frameworks employed, including Mantel tests, network analysis, and variance partitioning, identify patterns of co-variation rather than direct causality. Although contig-level co-localization provides supporting evidence for physical linkage, it does not demonstrate functional horizontal gene transfer rates or the strength of metal-driven co-selection in vivo. The hypothesized model, in which MGE-mediated horizontal gene transfer and MRG-related co-selection actively drive gut resistome assembly, awaits direct experimental validation. Future work should integrate multi-seasonal sampling with controlled laboratory experiments, such as in vitro conjugation assays and metal-exposure culturing of fish gut isolates, to establish causal relationships and incorporate long-read sequencing and Hi-C to improve host-tracking resolution.

## 5. Conclusions

This investigation systematically elucidated the structure, driving factors, and transmission potential of the gut resistome in three dominant wild fish species from the Qiantang River basin. The main conclusions are as follows: (1) Fish guts harbor a highly diverse repertoire of ARGs, but their composition exhibits marked host-species specificity, indicating that the gut environment functions as a selective filter for ARGs rather than a simple mirror of the environmental resistome. (2) MGEs and MRGs are the factors most closely associated with gut ARG variation, with their relative importance varying according to fish ecological habits, forming a dominant mobile-element-associated and co-selection-linked pattern. (3) Metagenomic assembly identified the physical linkage of ARG-MGE-MRG on both plasmids and chromosomes, providing supporting genomic structural evidence for the potential horizontal dissemination of ARGs. (4) Risk ranking revealed significant enrichment of Rank II potentially high-risk ARGs (e.g., *mcr* family, *blaZ*) in fish guts. Binning confirmed their carriage by potential pathogens such as *Staphylococcus*, while *Aeromonas* harbored mcr-7.1 on a plasmid. We recommend integrating risk-ranking-based surveillance into watershed monitoring, particularly targeting fish guts as high-risk niches for ARG dissemination through the food web.

While these findings are primarily correlational and based on a single-season survey (see Section 4.6 for detailed limitations), they provide a robust basis for recommending a management shift towards interrupting dissemination pathways. Future research incorporating long-read sequencing, time-series sampling, and functional validation could deepen investigations along three dimensions: precise host tracking, dynamic succession, and causal mechanisms. In summary, this study recommends that resistance pollution management in river basins be shifted from traditional concentration-based control toward strategies that interrupt dissemination pathways, adopting MGEs as potential early warning indicators while simultaneously strengthening the regulation of heavy metal pollution sources, such as industrial wastewater, to suppress the propagation of ARGs within food webs.

## Data Availability

The raw metagenomic sequencing data generated in this study have been deposited in the NCBI Sequence Read Archive (SRA) under BioProject accession number PRJNA1453778. Processed abundance tables for ARGs, MGEs, MRGs, and microbial taxa at the genus level are provided in Supplementary Table S6.

## Supplementary Materials

The following supporting information can be downloaded at: https://www.mdpi.com/article/10.3390/ani16132000/s1, Table S1a: Analytical performance parameters for antibiotic quantification by HPLC-MS/MS; Table S1b: Heavy metal analytical parameters by ICP-MS; Table S2: A comprehensive table of all Mantel test results (r and p-values); Table S3: Spearman’s rank correlations between ARGs and bacterial genera (AN, CN, MT), and between ARGs and MGEs/MRGs, with raw *p*-values and FDR-adjusted *q*-values.; Table S4: Annotations and localization predictions for all 2622 ARG-carrying contigs (ACCs); Table S5: Summary of ARG-carrying MAGs; Table S6: Abundance data for ARGs, MRGs, MGEs, and microbial community.
